# Prunes May Blunt Adverse Effects of Oral Contraceptives on Bone Health in Young Adult Women: A Randomized Clinical Trial

**DOI:** 10.1016/j.cdnut.2024.104417

**Published:** 2024-07-18

**Authors:** Taylor DeMasi, Michelle Tsang, Jenna Mueller, Kristine Giltvedt, Thuy Ngoc Nguyen, Mark Kern, Shirin Hooshmand

**Affiliations:** School of Exercise and Nutritional Sciences, San Diego State University, San Diego, CA, United States

**Keywords:** bone health, dried plums, oral contraceptives, women’s health, osteoporosis

## Abstract

**Background:**

Oral contraceptives (OCs) may promote bone loss, thereby leading to suboptimal bone health later in life. Research is needed to determine whether dietary interventions can blunt OC-related bone loss; prune consumption, shown to be effective in improving bone density in previous studies, could provide a safe and inexpensive solution.

**Objectives:**

The purpose of this study was to determine whether 12 mo of consuming 50 g of prunes daily would prevent bone loss or increase bone accrual in young (18–25 y) OC users.

**Methods:**

Ninety women were randomly assigned to a control group not using OCs (non-OC), an OC group not consuming prunes (OC), and an OC group consuming 50 g prunes daily (OC+P) for 12 mo. Bone mineral density (BMD) was measured at baseline and after 12 mo via dual-energy X-ray absorptiometry (at all sites) and peripheral quantitative computed tomography (at tibia). Blood samples were collected at baseline and after 6 and 12 mo to assess biomarkers.

**Results:**

There were no significant differences between groups for dietary intake, physical activity, serum tartrate-resistant acid phosphatase-5b, or bone alkaline phosphatase concentrations. Baseline serum C-reactive protein and vitamin D concentrations were higher (*P* < 0.001) for OC and OC+P groups than those in the non-OC group. Serum parathyroid hormone was higher for non-OC group than OC group at both baseline (*P* = 0.049) and final (*P* = 0.032). BMD measured by dual-energy X-ray absorptiometry at all sites did not change among groups. Ultradistal radius BMD increased over time (*P* < 0.05) within non-OC and OC+P groups. Trabecular density of the distal tibia as measured by peripheral quantitative computed tomography decreased from baseline to 12 mo within the OC group only (*P* = 0.016).

**Conclusions:**

OC use tended to promote minor negative effects on bone and consuming prunes tended to provide a potential protective effect on trabecular density of the distal tibia and ultradistal radius.

This trial was registered at www.clinicaltrials.gov as NCT04785131.

## Introduction

A large number of young adult women in their peak bone development years may be at increased risk for osteoporosis later in life owing to use of hormonal contraceptives [[1], [2], [3]]. It is estimated that two-thirds of young women using contraception use some form of hormonal contraceptives. Of hormonal contraceptive users, ∼68% use oral contraceptives (OCs) [4]. Most preparations of OCs include both ethinyl estradiol (EE) and progestin. Endogenous estrogen is recognized for enhancing the activity of osteoblasts and reducing osteoclastic activity, thereby promoting bone health [5]. In contrast, exogenous estrogen as provided by modern OC formulations has been demonstrated to promote loss of bone [6]. This occurs through suppression of endogenous estrogen production, which ultimately lowers circulating estradiol concentrations [[7], [8], [9]] with not only low-dose (20–35 μg EE) but also sometimes even lower dose preparations (<20 μg). Indeed, research has demonstrated that users of OCs have poorer bone health than nonusers [7,8,10].

Identifying nutritional interventions for OC users may be important to help prevent bone loss. Prunes are the most effective fruit in both preventing and reversing bone loss [11]. Bone-protective properties of prunes have been demonstrated in ovariectomized animal models of osteoporosis [12,13] and in postmenopausal women [[14], [15], [16]]. When effects of prunes were tested on young adult male mice with normal bones, prunes increased trabecular bone volume as a percent of total bone volume by nearly 50% above basal concentrations [17]. Although several animal studies have demonstrated bone-protective effects of prunes in young adult animals, no human study has evaluated the effect of prunes on bone metabolism in young humans. Although the mechanisms of action are not completely understood, research suggests that the effects of prunes occur primarily through inhibition of bone resorption by reducing the activity of osteoclasts [15,16]. Because OCs may promote bone loss by reducing bone turnover, we hypothesized that consumption of prunes would inhibit the potential adverse effects of OCs on bone.

## Methods

### Recruitment

This study was conducted at San Diego State Univerisity from December 2019 until October 2023. Potential participants were recruited for this study through flyers, classroom announcements, university e-mails, and online community outreach.

### Randomization

**FIGURE 1:**
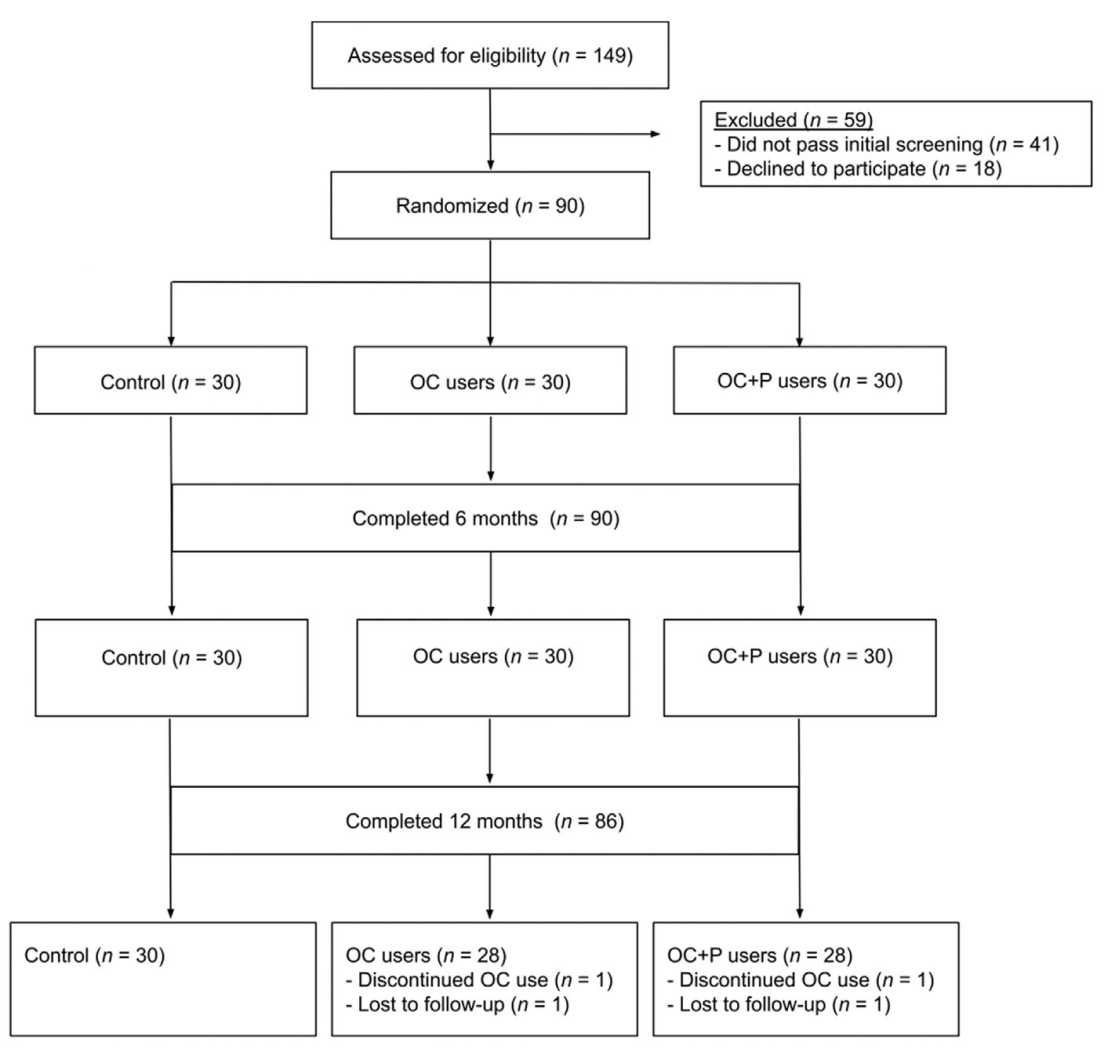
Flow diagram of enrollment and follow-up. OC, oral contraceptive; P, prune.

Participants were randomly allocated to groups using a computer-generated list of random numbers with 3 block sizes and a 1:1:1 group allocation ratio. Blinding to participant treatment assignment was not possible. Potential participants were given a questionnaire regarding their menstrual history and oral contraceptive use; based on their answers, participants were divided into a cohort using OCs (*n* = 60) and a cohort not using oral or other hormonal contraceptives (naturally cycling; *n* = 30). Participants using OCs were randomly assigned to either a group not consuming prunes and not provided a control food (OC; *n* = 30), or a group consuming 50 g prunes (OC+P; *n* = 30) daily for 12 mo (Figure 1).

### Study design

This study was a single-center, parallel-arm 12-mo randomized controlled trial. Ninety eligible healthy young females aged between 18 and 25 y (BMI: 18–32 kg/m^2^), not pregnant or lactating, nonsmokers, and consuming fewer than 2 alcoholic drinks per day were recruited. We also required that participants using OCs had used them for ≥1 y and no >5 y and those not using OCs had not used them for ≥3 mo before their enrollment. Complete medical history, a reproductive health survey, and dietary vitamin D and calcium questionnaires (short calcium and vitamin D questionnaire 2002) were obtained from participants before initiating the study. Participants receiving 50 g prunes/d were instructed to consume prunes as they preferred at any time of the day and either in divided doses or in their entirety. The amount of prunes was based on the findings of clinical trials that demonstrated that consumption of 50 g of prunes/d for 6 and 12 mo significantly improved bone density compared with a control group in postmenopausal women [16,18]. To avoid any potential laxative effects, participants were instructed to gradually increase their consumption of prunes in the span of 1 week culminating with consumption of 50 g/d. The composition of prunes per 50 g obtained from Food Processor SQL Software (version 10.12.0, 2012; ESHA Research) is as follows: 120 calories, 0.19 g fat, 31.5 g carbohydrate, 1.1 g protein, 3.55 g fiber, 21.5 mg calcium, 0.47 mg iron, 20.5 mg magnesium, 34.5 mg phosphorous, 366 mg potassium, 1 mg sodium, 0.22 mg zinc, 0.14 mg copper, 0.15 μg selenium, 19.5 μg vitamin A retinol activity equivalents, 197 μg β-carotene, and 29.75 μg vitamin K (phylloquinone). Participants were asked to record the days they missed consuming the study regimen. All participants provided written informed consent. We conducted this study in accordance with procedures approved by the San Diego State Institutional Review Board’s Human Research Protection Program.

### Dietary and physical activity assessment and anthropometric measurements

Height and weight were assessed at baseline and at 6 and 12 mo. Height was assessed using a wall-mounted stadiometer. Weight was measured with an electronic scale. The anthropometric data were used to calculate BMI (in kg/m^2^). The 2014 Block Food Frequency Questionnaire (FFQ; Berkeley Analytics) was administered at baseline and at 6 and 12 mo to assess dietary intake and physical activity over the study period. Participants were instructed to maintain their usual physical activity and diet pattern throughout the duration of the study.

### Serum bone biomarker measurements

Venous blood samples were obtained after an overnight fasting from each participant at baseline and after 6 and 12 mo of the study. Participants were asked to fast for 10 h before their scheduled blood draw visits, and OC users who consumed their pill in the morning were instructed to wait until after the blood draws. Blood samples were centrifuged at 1200 × *g* for 10 min at 4 °C, and serum samples were divided into aliquots and stored at −80 °C until analysis. Participants missing blood at any time point were not included in blood analyses for those time points only, resulting in some variation in sample sizes for some time points.

Bone alkaline phosphatase (BAP; Quidel), tartrate-resistant acid phosphatase (TRAP)-5b (Quidel), and C-reactive protein (CRP; Immundiagnostik AG) were analyzed by ELISA at all time points. 25-Hydroxy vitamin D (Alpco) and parathyroid hormone (PTH; Quidel) concentrations were analyzed at baseline and 12 mo. Intra-assay coefficient of variation was 5.2% for BAP, 3.0% for TRAP-5b, 7.3% for CRP, 3.4% for vitamin D, and 5.5% for PTH.

### Bone densitometry assessment

Bone density was assessed via dual-energy X-ray absorptiometry (DXA; GE Healthcare Lunar). Densitometer stability was evaluated by performance of phantom scans on the dates of all data acquisition. The precision of this technique, presented as the coefficient of variation, was 0.65% for the lumbar spine, 0.73% for the total hip, 0.85% for forearm, and 0.55% for total body locations. Scans were completed for analysis of bone density at the total body, lumbar spine (L1–L4), dual femur, and nondominant forearm at baseline and 12 mo for each participant. T scores only included participants who were aged ≥20 y at the time of scan.

### Peripheral quantitative computed tomography measurements

A single operator conducted peripheral quantitative computed tomography (pQCT) measurements (XCT-3000; Stratec Medizintechnik) of the left tibia. At the start of each testing day, a cortical and cone bone phantom (Bone Diagnostic LLC) specific to the pQCT with known properties was scanned and the variation in phantom measures differed by <1% to verify the calibration of the XCT-3000. The operator performed scout view scans of the distal left tibia and established a reference point at the end plate.

A single tomographic slice at the 4%, 38%, and 66% sites relative to the distal growth plate was obtained. Tibial length was measured to the nearest millimeter from the tibial plateau to the medial malleolus, with measurements done at 4%, 38%, and 66% of tibial length from the distal endplate. All sites were scanned with the following parameters: 0.4 mm pixel size and 20 mm/s scan speed. All sites were scanned at baseline and after 12 mo of intervention. The Stratec XCT 6.00 software package was used for image analysis. At all sites, a region of interest was drawn to isolate the tibia. The 4% site was analyzed with a threshold of 169 mg/cm^3^ and contour mode 3 and then peel mode 4 with an inner threshold of 650 mg/cm^3^ and a 10% concentric peel to define trabecular. The distal sites of 38% and 66% used a threshold of 710 mg/cm^3^ and cort mode 2 to define cortical bone for area and density measures. In addition, the distal sites were analyzed with cort mode 2 and a threshold of 480 mg/cm^3^ to determine the stress–strain index. Muscle parameters were assessed at the left tibia using a 0.8-mm pixel size. Measurements included intermuscular adipose tissue (square centimeters), muscle area (square centimeters), and muscle density (milligrams per cubic centimeter).

### Statistical analysis

Data were analyzed with JASP (version 0.18; JASP Team; 2023). Participants' baseline characteristics and baseline measurements were analyzed through a 1-way analysis of variance (ANOVA). Differences in DXA variables, pQCT variables, vitamin D, and PTH were determined using 3 (trial) × 2 (time) repeated-measures ANOVA. BAP, TRAP-5b, and CRP were determined using 3 (trial) × 3 (time) repeated-measures ANOVA. Because age at menarche was significantly different at baseline, we included it as a covariate in DXA, pQCT, and bone resorption and formation biomarkers. Variables without normal distribution were log transformed and outliers removed if normality was not established following log transformation. Friedman nonparametric *t* tests were applied if data were still not normally distributed. If variables violated sphericity, the Greenhouse–Geisser correction was applied. Follow-up Tukey post hoc tests and paired-samples and independent-samples *t* tests were used to examine statistical significance between and within the different time points for all markers. *A priori* sample sizes of 30 participants per group provided 80% power and an α level of 0.05, accounting for an attrition rate of 13%. Data are reported as means ± SD. Significance was selected at an α level of 0.05.

## Results

### Baseline characteristics, anthropometrics, dietary intake, and physical activity

Data from 86 women (30 not using oral contraceptives, 28 using oral contraceptives, and 28 using oral contraceptives and consuming 50 g prunes) who participated in the study were analyzed. Within both OC and OC+P groups, 1 participant stopped using OCs and 1 participant was lost to follow-up. There were no significant differences in baseline age, height, weight, BMI, mean days between periods, age of first OC use, alcohol consumption, or calcium and vitamin D intake between groups. The control (non-OC) group had a significantly lower age at menarche (*P* < 0.001) and significantly lower lifetime duration of OC use (*P* < 0.001) than both the OC and OC+P groups (Table 1). The OC+P group had a total compliance rate for prune consumption of 87%. Unlike both OC groups, more non-OC participants identified as Asian and Hispanic and none as Black or Other. The majority of all participants were White females. There were no significant differences between groups for macronutrient, vitamin, or mineral intake. Non-OC users consumed significantly less fiber (*P* < 0.001), vitamin K (*P* = 0.048), and folate (*P* = 0.02) over time (Table 2), although intake of vitamin K and folate remained above the recommended dietary allowances. With regard to fiber, baseline intake of all groups started below the recommended dietary allowance but dropped even further below that level within the non-OC group. Additionally, there were no significant differences for total energy expenditure.

**TABLE 1:** Participant characteristics at study entry^1^

Variable	Non-OC (*n* = 30)	OC (*n* = 28)	OC+P (*n* = 28)	*P*
Age (y)	21.4 ± 1.9	20.9 ± 1.6	21.5 ± 1.9	0.398
Height (cm)	161.4 ± 5.7	164.2 ± 5.5	164.8 ± 6.4	0.071
Weight (kg)	60.9 ± 11.2	61.0 ± 7.2	61.9 ± 12.4	0.936
BMI (kg/m^2^)	23.3 ± 3.4	22.7 ± 2.5	22.7 ± 4.0	0.734
Age at menarche (y)	11.9 ± 1.5ᵃ	13.3 ± 1.1ᵇ	13.1 ± 1.6ᵇ	<0.001
Average days between periods (d)	28.0 ± 3.5	26.0 ± 3.1	27.2 ± 2.9	0.073
Age of first OC use (y)	17.9 ± 2.4	17.4 ± 1.6	17.1 ± 2.0	0.455
Duration of OC use (y)	1.0 ± 1.6ᵃ	3.3 ± 2.2ᵇ	4.0 ± 2.2ᵇ	<0.001
Duration of alcohol use (y)	2.7 ± 2.6	2.4 ± 1.8	3.6 ± 2.0	0.105
Calcium intake (mg/d)	922 ± 417	1020 ± 501	958 ± 385	0.694
Vitamin D intake (IU/d)	545 ± 831	447 ± 983	446 ± 1035	0.900
Race (%)				
Asian	23	14	11	—
Black	0	7	4	—
Hispanic	33	21	7	—
White	43	50	71	—
Other	0	7	7	—

Abbreviations: OC, oral contraceptive; P, prune.

*P* value for ANOVA. *P* value α level <0.05.

ᵃᵇᶜWithin a row, means without a common superscript differ (*P* < 0.05).

1 Values are means ± SDs.

**TABLE 2:** Dietary intake and physical activity at baseline, 6 mo, and 12 mo^1^

Variable	Non-OC (*n* = 29)			OC (*n* = 26)			OC+P (*n* = 25)			*P*	*P*	*P*
	Baseline	6 mo	12 mo	Baseline	6 mo	12 mo	Baseline	6 mo	12 mo	Time × treatment	Time	Treatment
Macronutrients												
Calories (kcal)	1596 ± 653	1433 ± 455	1441 ± 443	1502 ± 682	1388 ± 491	1453 ± 658	1575 ± 502	1607 ± 561	1646 ± 752	0.54	0.38	0.49
Protein (g)	61 ± 26	56 ± 25	55 ± 17	62 ± 30	56 ± 25	58 ± 28	64 ± 24	62 ± 25	64 ± 32	0.90	0.31	0.58
Carbohydrates (g)	188 ± 82	159 ± 64	160 ± 63	169 ± 81	154 ± 63	162 ± 83	178 ± 55	186 ± 60	188 ± 86	0.18	0.23	0.43
Fat (g)	67 ± 30	64 ± 23	64 ± 23	63 ± 29	58 ± 21	62 ± 27	66 ± 23	65 ± 25	68 ± 33	0.86	0.48	0.68
Fiber (g)	20 ± 10	16 ± 9∗	16 ± 9∗	16 ± 8	15 ± 7	14 ± 7	18 ± 6	18 ± 7	17 ± 9	0.09	0.003	0.29
Vitamins												
Vitamin A (μg)	747 ± 392	664 ± 305	698 ± 316	750 ± 410	662 ± 355	709 ± 451	665 ± 293	754 ± 392	667 ± 340	0.33	0.71	0.99
Vitamin E (mg)	9 ± 4	7 ± 4	8 ± 5	8 ± 4	7 ± 4	8 ± 5	8 ± 4	9 ± 4	9 ± 4	0.39	0.87	0.6
Vitamin D (IU)	160 ± 120	200 ± 120	160 ± 80	160 ± 120	160 ± 160	200 ± 160	160 ± 80	120 ± 80	160 ± 80	0.61	0.70	0.23
Vitamin K (μg)	223 ± 204	152 ± 114∗	167 ± 141∗	174 ± 142	137 ± 100	136 ± 103	159 ± 114	146 ± 94	159 ± 89	0.37	0.012	0.51
Vitamin C (mg)	92 ± 67	77 ± 44	80 ± 50	77 ± 52	74 ± 54	69 ± 48	74 ± 44	76 ± 45	84 ± 51	0.46	0.64	0.69
Vitamin B-1 (mg)	1.4 ± 0.6	1.2 ± 0.5	1.2 ± 0.5	1.3 ± 0.6	1.1 ± 0.6	1.2 ± 0.6	1.4 ± 0.5	1.3 ± 0.4	1.3 ± 0.7	0.81	0.08	0.51
Vitamin B-2 (mg)	1.5 ± 0.6	1.5 ± 0.5	1.5 ± 0.5	1.5 ± 0.7	1.4 ± 0.8	1.4 ± 0.9	1.6 ± 0.7	1.6 ± 0.6	1.7 ± 0.9	0.86	0.88	0.40
Vitamin B-3 (mg)	19 ± 8	17 ± 5	16 ± 5	19 ± 8	17 ± 8	17 ± 9	20 ± 8	20 ± 7	20 ± 11	0.56	0.22	0.15
Vitamin B-6 (mg)	1.7 ± 0.7	1.5 ± 0.5	1.5 ± 0.6	1.7 ± 0.8	1.4 ± 0.7	1.4 ± 0.8	1.8 ± 0.8	1.8 ± 0.7	1.9 ± 1.1	0.58	0.19	0.12
Folate (μg)	489 ± 225	407 ± 162∗	411 ± 177∗	439 ± 235	388 ± 207	379 ± 198	456 ± 155	448 ± 131	438 ± 186	0.69	0.03	0.54
Vitamin B-12 (μg)	3 ± 2	4 ± 2	3 ± 1	4 ± 2	3 ± 2	4 ± 2	4 ± 2	4 ± 2	4 ± 2	0.78	0.96	0.46
Choline (mg)	259 ± 104	245 ± 100	241 ± 70	252 ± 111	236 ± 111	244 ± 111	256 ± 99	252 ± 106	251 ± 111	0.98	0.55	0.93
Minerals												
Calcium (mg)	792 ± 318	772 ± 243	785 ± 287	838 ± 373	805 ± 489	866 ± 491	786 ± 230	795 ± 289	838 ± 356	0.95	0.54	0.81
Iron (mg)	13 ± 5	11 ± 4	11 ± 4	12 ± 6	10 ± 5	11 ± 6	12 ± 4	13 ± 5	12 ± 5	0.32	0.17	0.38
Magnesium (mg)	300 ± 124	262 ± 99	259 ± 100	262 ± 115	236 ± 116	254 ± 140	272 ± 85	286 ± 95	287 ± 128	0.17	0.27	0.51
Phosphorus (mg)	1109 ± 456	1025 ± 324	1019 ± 355	1112 ± 478	1021 ± 467	1103 ± 578	1112 ± 354	1110 ± 420	1149 ± 527	0.74	0.39	0.79
Potassium (mg)	2365 ± 1106	2104 ± 808	2107 ± 870	2072 ± 869	1902 ± 821	1990 ± 1049	2130 ± 645	2202 ± 740	2235 ± 947	0.42	0.37	0.55
Sodium (mg)	2884 ± 1284	2477 ± 932	2467 ± 907	2742 ± 1225	2614 ± 1019	2522 ± 1093	2800 ± 1020	2667 ± 1031	2731 ± 1376	0.68	0.08	0.88
Zinc (mg)	9 ± 4	8 ± 4	8 ± 3	9 ± 4	8 ± 4	8 ± 5	9 ± 3	9 ± 3	9 ± 4	0.91	0.31	0.48
Copper (mg)	1.3 ± 0.5	1.2 ± 0.5	1.1 ± 0.4	1.2 ± 0.5	1.1 ± 0.5	1.2 ± 0.6	1.2 ± 0.4	1.3 ± 0.4	1.3 ± 0.6	0.12	0.42	0.52
Selenium (μg)	83 ± 37	77 ± 30	74 ± 22	87 ± 42	79 ± 34	81 ± 38	88 ± 36	83 ± 33	86 ± 42	0.93	0.2	0.6
Physical activity												
Total EE (kcal)	604 ± 466	640 ± 522	557 ± 522	727 ± 474	695 ± 447	737 ± 471	669 ± 450	754 ± 562	1194 ± 1667	0.12	0.21	0.18

Abbreviations: EE, energy expenditure; OC, oral contraceptive; P, prune.

*P* value for repeated-measures ANOVA. *P* value α level <0.05.

1 Values are means ± SDs. Analysis did not include 50 g prunes/d consumed by the OC+P group.

∗ Significantly different from baseline within a trial (*P* < 0.05).

### Serum bone biomarkers

Serum concentrations of bone biomarkers including TRAP-5b and BAP did not change within or between groups (Table 3). Those not using OCs had lower (*P* < 0.001) CRP at all time points than OC users and at baseline and 12 mo in comparison with OC+P users. Vitamin D concentrations were significantly higher at baseline in OC and OC+P groups than those in the non-OC group (*P* < 0.001). Additionally, OC+P users tended to have lower vitamin D concentrations at 12 mo than baseline values (*P* = 0.051). PTH concentrations were significantly higher in non-OC users at baseline (*P* = 0.049) and final (*P* = 0.032) than those in the OC group.

**TABLE 3:** Comparison of serum biomarkers^1^

Variable	Non-OC (*n* = 27)			OC (*n* = 26)			OC+P (*n* = 24)			*P*	*P*	*P*
	Baseline	6 mo	12 mo	Baseline	6 mo	12 mo	Baseline	6 mo	12 mo	Time × treatment	Time	Treatment
BAP (U/L)	22.73 ± 6.59	22.07 ± 6.05	22.29 ± 6.73	22.48 ± 6.40	22.59 ± 9.07	20.96 ± 6.14	20.88 ± 7.39	20.75 ± 4.65	19.34 ± 5.39	0.882	0.940	0.351
TRAP-5b (U/L)	3.05 ± 1.03	2.90 ± 0.90	2.86 ± 0.93	3.25 ± 1.14	3.18 ± 1.04	2.99 ± 0.90	3.47 ± 1.29	3.41 ± 1.26	3.25 ± 1.02	0.878	0.637	0.408
CRP (mg/L)	2.44 ± 3.92ᵃ	2.89 ± 5.10ᵃ	2.10 ± 2.83ᵃ	8.26 ± 5.98ᶜ	8.49 ± 7.14ᵇ	6.79 ± 6.64ᵇ	4.69 ± 3.58ᵇ	5.59 ± 5.69ᵃᵇ	6.36 ± 5.63ᵇ	0.288	0.046	0.145
Vitamin D (ng/mL)	23.0 ± 13.2ᵃ	—	22.4 ± 10.0ᵃ	35.3 ± 12.6ᵇ	—	33.2 ± 11.1ᵇ	40.4 ± 17.2ᵇ	—	37.3 ± 15.3ᵇ^,^∗	0.456	<0.001	0.456
PTH (pg/mL)	36.6 ± 13.1ᵃ	—	34.9 ± 13.4ᵃ	29.2 ± 14.2ᵇ	—	26.9 ± 13.7ᵇ	32.6 ± 12.9ᵃᵇ	—	28.4 ± 12.3ᵃᵇ	0.804	0.094	0.036

Abbreviations: BAP, bone alkaline phosphatase; CRP, C-reative protein; OC, oral contraceptive; P, prune; PTH, parathyroid hormone; TRAP-5b, tartrate-resistant acid phosphatase-5b.

*P* value for repeated-measures ANOVA. *P* value α level <0.05.

ᵃᵇᶜDifferent superscripts indicate difference within timepoints between trials (*P* < 0.05).

1 Values are means ± SDs.

∗ Significantly different from baseline within a trial (*P* < 0.05).

### Bone mineral density via DXA

There were no significant differences between or within groups for bone mineral density (BMD) at the lumbar spine, right femur, left femur, nondominant radius and ulna, or total body as well as total body T score (Table 4). However, BMD at the nondominant ultradistal radius increased significantly from baseline to 12 mo in non-OC [*P* = 0.003; effect size: 0.595 (0.202, 0.979)] and OC+P [*P* = 0.020; effect size: 0.468 (0.074, 0.855)] groups.

**TABLE 4:** Comparison of bone mineral density, T scores, and pQCT variables for various bone sites^1^

Variable	Non-OC (*n* = 30)		OC (*n* = 28)		OC+P (*n* =28)		*P*	*P*	*P*
	Baseline	12 mo	Baseline	12 mo	Baseline	12 mo	Time × treatment	Time	Treatment
DXA									
Total body BMD (g/cm^2^)	1.174 ± 0.071	1.178 ± 0.073	1.183 ± 0.069	1.187 ± 0.073	1.188 ± 0.079	1.188 ± 0.081	0.583	0.570	0.944
Total body T score	0.710 ± 0.905	0.765 ± 0.917	0.756 ± 0.904	0.823 ± 0.969	0.774 ± 1.085	0.769 ± 1.094	0.459	0.950	0.996
Lumbar spine BMD (g/cm^2^)	1.214 ± 0.126	1.221 ± 0.119	1.257 ± 0.113	1.258 ± 0.115	1.229 ± 0.120	1.235 ± 0.122	0.630	0.723	0.625
Right femur BMD (g/cm^2^)	1.016 ± 0.136	1.016 ± 0.132	1.058 ± 0.121	1.059 ± 0.123	1.086 ± 0.118	1.084 ± 0.118	0.846	0.904	0.309
Left femur BMD (g/cm^2^)	1.022 ± 0.131	1.020 ± 0.123	1.065 ± 0.118	1.063 ± 0.118	1.076 ± 0.113	1.072 ± 0.115	0.936	0.626	0.578
Forearm BMD (g/cm^2^)	0.610 ± 0.047	0.621 ± 0.044	0.616 ± 0.047	0.616 ± 0.046	0.610 ± 0.066	0.619 ± 0.072	0.210	0.868	0.977
Radius UD BMD (g/cm^2^)	0.435 ± 0.050	0.450 ± 0.044∗	0.449 ± 0.053	0.447 ± 0.057	0.446 ± 0.070	0.459 ± 0.075∗	0.047	0.823	0.593
Ulna UD BMD (g/cm^2^)	0.321 ± 0.047	0.331 ± 0.052	0.316 ± 0.040	0.323 ± 0.051	0.311 ± 0.052	0.317 ± 0.066	0.744	0.432	0.761
pQCT									
4% Tibia									
Total density (mg/cm^3^)	331.3 ± 46.9	328.6 ± 43.8	341.5 ± 53.6	337.2 ± 51.0	324.0 ± 42.2	317.7 ± 41.2	0.838	0.413	0.347
Total area (mm^2^)	906.2 ± 132.4	896.4 ± 120.9	891.4 ± 124.2	903.2 ± 120.4	924.0 ± 143.8	944.2 ± 131.5	0.400	0.465	0.464
Trabecular density (mg/cm^3^)	266.4 ± 50.1	260.3 ± 46.0	280.6 ± 58.6	273.7 ± 47.8∗	254.7 ± 41.7	254.7 ± 36.8	0.135	0.001	0.351
Trabecular area (mm^2^)	718.0 ± 114.0	709.3 ± 107.5	704.6 ± 115.8	710.4 ± 107.2	734.8 ± 136.3	756.3 ± 123.9	0.232	0.556	0.370
SSI (mm^3^)	2130.0 ± 448.6	2141.7 ± 392.2	2206.1 ± 519.6	2213.5 ± 541.4	2195.9 ± 395.4	2178.7 ± 406.1	0.845	0.712	0.916
38% Tibia									
Total content (mg/mm)	330.9 ± 42.5	331.1 ± 46.9	333.0 ± 35.2	333.9 ± 33.9	334.4 ± 42.8	335.5 ± 42.6	0.745	0.161	0.966
Total density (mg/cm^3^)	951.5 ± 50.7	955.6 ± 44.0	937.6 ± 44.4	941.3 ± 44.1	948.5 ± 44.9	947.5 ± 46.4	0.559	0.611	0.584
Total area (mm^2^)	348.7 ± 47.6	356.5 ± 57.9	355.2 ± 34.3	354.7 ± 31.9	355.4 ± 41.7	357.1 ± 42.1	0.208	0.384	0.935
Cortical content (mg/mm)	316.0 ± 41.6	313.9 ± 51.8	318.0 ± 34.3	318.8 ± 33.2	319.6 ± 43.7	320.3 ± 42.2	0.542	0.136	0.974
Cortical density (mg/cm^3^)	1181.5 ± 28.4	1184.3 ± 34.1	1186.8 ± 25.3	1190.2 ± 25.4	1186.4 ± 15.8	1186.6 ± 21.6	0.883	0.395	0.211
Cortical area (mm^2^)	267.5 ± 35.7	265.2 ± 45.3	268.1 ± 30.1	267.9 ± 28.2	269.5 ± 37.6	270.1 ± 37.2	0.516	0.166	0.959
Cortical thickness (mm)	5.5 ± 0.5	5.5 ± 0.5	5.4 ± 0.5	5.4 ± 0.5	5.5 ± 0.5	5.5 ± 0.5	0.929	0.698	0.619
Periosteal circumference (mm)	66.1 ± 4.5	66.7 ± 5.4	66.7 ± 3.2	66.7 ± 3.0	66.7 ± 3.9	66.9 ± 3.9	0.242	0.429	0.969
Endosteal circumference (mm)	31.7 ± 3.8	31.8 ± 3.8	33.0 ± 2.8	32.9 ± 2.9	32.7 ± 3.8	32.9 ± 3.8	0.362	0.983	0.465
SSI (mm^3^)	1422.5 ± 290.9	1412.5 ± 304.3	1452.6 ± 213.2	1456.1 ± 201.3	1461.8 ± 264.7	1476.7 ± 266.4	0.444	0.169	0.927
66% Tibia									
Total content (mg/mm)	341.9 ± 47.4	338.7 ± 48.0	352.8 ± 37.8	352.7 ± 35.4	354.0 ± 46.0	355.3 ± 45.5	0.278	0.133	0.122
Total density (mg/cm^3^)	614.7 ± 91.9	603.1 ± 89.3	619.8 ± 79.9	621.1 ± 81.8	643.7 ± 79.7	645.2 ± 71.5	0.210	0.990	0.258
Total area (mm^2^)	562.4 ± 80.0	567.8 ± 80.9	573.3 ± 57.3	572.2 ± 55.3	554.5 ± 74.0	554.2 ± 71.0	0.978	0.196	0.580
Cortical content (mg/mm)	293.5 ± 49.5	288.9 ± 49.6	307.1 ± 41.2	307.6 ± 39.5	307.6 ± 41.6	309.4 ± 41.0	0.261	0.355	0.490
Cortical density (mg/cm^3^)	1124.1 ± 27.8	1123.0 ± 27.7	1131.0 ± 24.4	1134.8 ± 25.0	1132.6 ± 26.8	1134.3 ± 19.4	0.792	0.838	0.194
Cortical area (mm^2^)	260.8 ± 42.1	257.1 ± 42.7	271.5 ± 35.2	270.9 ± 32.6	271.6 ± 36.4	272.8 ± 36.0	0.254	0.280	0.618
Cortical thickness (mm)	3.6 ± 0.7	3.5 ± 0.7	3.7 ± 0.6	3.7 ± 0.6	3.8 ± 0.5	3.8 ± 0.5	0.164	0.648	0.397
Periosteal circumference (mm)	83.9 ± 6.0	84.2 ± 6.2	84.2 ± 4.2	84.7 ± 4.1	83.3 ± 5.7	83.3 ± 5.5	0.990	0.190	0.572
Endosteal circumference (mm)	61.0 ± 8.2	62.0 ± 8.0	61.2 ± 6.8	61.2 ± 6.9	60.0 ± 5.6	59.9 ± 5.1	0.610	0.410	0.676
SSI (mm^3^)	2087.6 ± 396.7	2083.7 ± 399.8	2190.9 ± 319.0	2188.6 ± 294.1	2141.4 ± 396.7	2174.4 ± 392.3	0.449	0.138	0.936
Muscle									
IMAT (cm^2^)	1435.9 ± 306.0	1413.5 ± 347.5	1399.7 ± 211.0	1423.9 ± 214.5	1405.3 ± 317.1	1407.1 ± 258.9	0.568	0.623	0.872
Area (cm^2^)	5825.5 ± 940.4	5646.2 ± 1173.9	5792.6 ± 576.6	5778.5 ± 549.8	5726.0 ± 841.3	5800.1 ± 784.8	0.457	0.138	0.637
Density (mg/cm^3^)	81.2 ± 1.7	80.9 ± 1.9	81.3 ± 1.3	80.8 ± 1.2	80.3 ± 1.3	80.7 ± 1.3	0.160	0.367	0.083

Abbreviations: BMD, bone mineral density; DXA, dual-energy X-ray absorptiometry; IMAT, intermuscular adipose tissue; OC, oral contraceptive; P, prune; pQCT, peripheral quantitative computed tomography; SSI, stress–strain index; UD, ultradistal.

*P* value for repeated-measures ANOVA. *P* value α level <0.05.

1 Values are means ± SDs.

∗ Significantly different from baseline within a trial (*P* < 0.05).

### Peripheral quantitative computed tomography

The 4%, 38%, and 66% sites of the tibia did not show significant difference between or within groups for total density, total area, trabecular area, total content, cortical content, cortical density, cortical area, cortical thickness, periosteal circumference, endosteal circumference, or stress–strain index (Table 4). Trabecular density at the 4% site did not follow a normal distribution and a Friedman nonparametric test revealed a main effect for time (*P* = 0.001) for decreasing density from baseline to final, and follow-up paired *t* tests indicated that the decrease was primarily within the OC group [*P* = 0.016; effect size: 0.508 (0.094, 0.912)]. There were no significant differences between or within groups for intermuscular adipose tissue, muscle area, or muscle density (Table 4).

## Discussion

The bone health measurements within this study provide evidence that consumption of prunes inhibits minor adverse effects of OCs on bone. These results suggest that to a limited degree, prunes may be therapeutic against OC-induced bone loss. Specifically, trabecular density of the distal tibia decreased over time only among individuals using OCs who were not consuming prunes, suggesting that prune intake may have blunted this effect in OC users. Koltun et al. [19] observed a similar decrease in trabecular density at the 4% site of the tibia in the control group compared with no change in the group consuming 50/100 g prunes (pooled) daily in postmenopausal women. Furthermore, in line with previous reports [7,20], OC use tended to blunt promotion of bone deposition that was detected in the ultradistal radius of the non-OC group as exhibited by a 3.44% increase over 12 mo. Interestingly, OC+P increased BMD (2.4%) at the ultradistal radius, which is also a highly trabecular bone site prone to fracture in women [21] while OC only maintained the BMD at this bone site. Because no other differences or changes in DXA or pQCT measures were detected, the effects within this timeframe appear to be limited. With regard to bone biomarkers, no differences in BAP or TRAP-5b were detected; however, the OC groups had significantly higher CRP and vitamin D concentrations at baseline, suggesting that regular use of OCs may put individuals at risk for inflammatory responses, yet improve vitamin D status, which may have effects that counter one another regarding most markers of bone health. Results from previous studies have shown that combined hormonal contraceptives containing both estrogens and progestins may increase serum concentrations of vitamin D depending on the formulations of the OCs [22,23]. Although the mechanism remains to be determined, research should further examine the effects of OCs on vitamin D status in young adult OC users. Furthermore, PTH concentrations in the non-OC group were higher than those in the OC group at baseline, which may also have implications for bone health measurements [24].

Previous research has suggested that timing of first OC use relative to menarche may affect BMD [25,26]. It is possible that initiation of OCs within 4 y of menarche can lower BMD at certain sites than those who initiate OC use >4 y after menarche [1]. Because both OC and OC+P participants in this study initiated OC use ∼4 y after menarche, the total duration (limited to no >5 y of continuous use before the start of the study) of OC use following menarche in the OC and OC+P groups may have been insufficient for producing greater detriments to bone health than we observed, which may have limited the capacity of a prune intervention to produce more robust results.

Non-OC users had a lower age at menarche than both OC and OC+P groups in this study, which may have confounded our results. Although the influence that age at menarche exerts on bone remains debated, higher age at menarche (>16 y of age) has been linked with lower peak bone mass and lower age at menarche (<12 y of age) is associated with higher peak bone mass in women [27,28]. The mean menarchal age of 11.9 y in non-OC users may have benefited bone accrual of those participants, given non-OC users exhibited increased BMD whearas OC users did not at the nondominant ultradistal radius over the duration of the study. Furthermore, because neither of the OC groups had mean ages at menarche older than 16 y, it is possible that their relatively young ages at menarche of ∼13 y prevented detection of even poorer bone health in comparison with the non-OC group.

Importantly, OC dosage may have an impact on bone turnover, and the OC preparations taken by our participants varied in dosage, which may have increased the variability of our outcomes. Researchers hypothesize that very low-dose OCs (≤20 μg EE) may decrease bone density even more than what are considered to be typical low-dose OCs (>20 μg EE) [7]. OC+P had a distribution of approximately half very low and half low-dose users, whereas the OC group included two-thirds low-dose users and one-third very lose dose users. If very low-dose OCs are indeed more detrimental to bone as hypothesized, it is possible that the inclusion of more very low-dose users in the OC+P group masked our capacity to detect some potential bone-protective impacts of prunes.

The decrease (2.45%) in trabecular density of the distal tibia among the OC participants who did not consume prunes may indicate some protective effects of prunes specifically on trabecular bone. Trabecular bone is particularly important for bone health, because skeletal sites with more trabecular bone are those that are most prone to fracture due to osteoporosis [29,30]. This may have important repercussions despite no other major bone health outcomes. Hooshmand et al. [15,16] and De Souza et al. [18] have demonstrated the capacity for prunes to preserve bone at sites that are known to have high degrees of trabecular bone in postmenopausal women.

The within-group difference in trabecular density of the distal tibia for the OC group not consuming prunes cannot be attributed to other differences in dietary intake or physical activity. The results from the FFQs showed no between-group differences in the background diets of participants for macronutrients, vitamins or minerals, as well as for total energy expenditure. Although no differences were detected, it should be noted that FFQ measurements are susceptible to error due to closed-ended format of the method and likelihood of recall bias, although it has been suggested to be the most advanced method for dietary recall [31]. With regard to measurement of physical activity, a bone-specific physical activity questionnaire [32] may have helped determine whether activity confounded our results.

Higher CRP concentrations in both OC groups are congruent with results from other studies showing OCs increase inflammatory markers [33,34]. Elevated CRP concentrations are associated with decreased BMD owing to its impact on bone resorption [35], which may be linked to the observed decrease in trabecular density of the distal tibia. Furthermore, as mentioned earlier, the higher vitamin D status of OC and OC+P may have blunted overall negative impacts of OCs on bone.

One of the limitations of this study was variation in OC formulations. Because participants were not administered the same OC pill type, the differences in progesterone may have contributed to varied impacts on bone [36]. Individuals in this population potentially also had varied peak bone development. Including bone age parameters in this study could have been beneficial to confirm that bone development did not differ between participants [37]. Additionally, a limitation of our research was a potential low compliance rate. Prune consumers were instructed to report any unconsumed prune packets every 2 wk. Of the responses, the total compliance rate was 87%; however, responses were only provided ∼50% of the time requested. Lower than prescribed prune consumption may have limited our ability to detect the impacts of the intervention.

Prunes have been shown to promote bone health in animal studies and to prevent bone loss in postmenopausal women in past research. The results from this study provide evidence that similar benefits can be detected in young adult women using OCs. Future research should better control for potential confounders such as age at menarche and duration and doses of OCs used to better evaluate the capacity of prunes to prevent aberrant effects of OCs on bone.

In conclusion, in this study, OC use was linked to minor detriments on bone and consumption of prunes may have mitigated the deleterious effects of OCs. Factors such as differences in age at menarche between treatment groups and doses of OCs used may have limited these findings. Future research should evaluate bone health in users of OCs with early life OC initiation following menarche and include an additional bone remodeling cycle for a total trial length of 24 mo to assess the impact of long-term OC use for early initiators and the potential for prune consumption to blunt their adverse effects.

## Acknowledgments

We gratefully acknowledge the valuable assistance in data collection that we received from the following students at San Diego State University: Jenna Laughlin, Jeff Moore, and Svitlana Storm.

## Author contributions

The authors’ responsibilities were as follows – SH, MK: designed the research; KG, TNN, MT, TD, SH: collected the the data; TD, JM: analyzed data; TD, MK, SH: interpreted the findings; TD, MT, SH, MK: wrote the article; SH: had primary responsibility for final content; and all authors: read and approved the final manuscript.

## Conflict of interest

The authors report no conflict of interest.

## Funding

This study was funded by the California Prune Board. The California Prune Board had no role in the design and conduct of the study; collection, management, analysis, and interpretation of the data; preparation, review, or approval of the manuscript; and decision to submit the manuscript for publication.

## Data availability

Data described in the manuscript will be made available upon request and pending approval.
